# Electrochemical Synthesis of Functionalized Graphene/Polyaniline Composite Using Two Electrode Configuration for Supercapacitors

**DOI:** 10.3390/nano13243140

**Published:** 2023-12-14

**Authors:** Dongsheng Yu, Jili Li, Tiekun Jia, Binbin Dong, Zhixiao Han, Wenjie Tian, Ruilin Jiang, Xi Lu, Lekang Li

**Affiliations:** Henan Province International Joint Laboratory of Materials for Solar Energy Conversion and Lithium Sodium Based Battery & Henan Key Laboratory of Special Protective Materials, Material Science and Engineering School, Luoyang Institute of Science and Technology, Luoyang 471023, China; tiekun_jia@lit.edu.cn (T.J.); dongbb@lit.edu.cn (B.D.); zhixiaohan2023@163.com (Z.H.); tianwenjie0305@163.com (W.T.); jiang_ruilin@163.com (R.J.); luxi20031101@163.com (X.L.); lekangli2003@163.com (L.L.)

**Keywords:** aryl diazonium salt-functionalized graphene (ADS-G), ADS-G/PANI composites, electrochemical polymerization, supercapacitor

## Abstract

An effective approach for the large-scale fabrication of conducting polyaniline (PANI) using in situ anodic electrochemical polymerization on nickel foam which had been coated in aryl diazonium salt (ADS)-modified graphene (ADS-G). In the present work, ADS-G was used as a high surface-area support material for the electrochemical polymerization of PANI. The electrochemical performances of the ADS-G/PANI composites exhibited better suitability as supercapacitor electrode materials than those of the PANI. The ADS-G/PANI composites achieved a specific capacitance of 528 F g^−1^, which was higher than that of PANI (266 F g^−1^) due to excellent electrode–electrolyte interaction and the synergistic effect of electrical conductivity between ADS-G and PANI in the composites. These findings suggest that the ADS-G/PANI composites are a suitable composite for potential supercapacitor applications.

## 1. Introduction

Since the first fabrication of graphene in its true sense, in 2004 [1], in a flat single layer of carbon atoms tightly packed into a two-dimensional honeycomb lattice [2,3,4,5], it has been an attractive substrate material to compound with a polymer for new energy storage, like the supercapacitor, due to its high conductivity and large surface area [6]. On the other hand, conducting polymers (CPs) such as polyaniline (PANI), polypyrrole (PPy), polythiophene (PT) have also been widely explored for supercapacitor use in the scientific community [7,8,9], as they provide conductivity through a conjugated bond system along the polymer backbone [10]. Due to their high flexibility and relatively high specific capacitance, CPs are considered one of the major electrode materials for supercapacitor applications. The combination of CPs with graphene is expected to engender some fascinating properties in view of the fascinating effects that CPs normally introduce into a nanostructure to improve electrochemical performance [11]. Yan et al. synthesized graphene nanosheet (GNS)/PANI composites using in situ polymerization [12]. Another effective approach for the preparation of PPy/graphene nanocomposites used graphite oxide and a pyrrole monomer via in situ polymerization which was subsequently reduced by hydrazine monohydrate [13]. Shi et al. reported that the sulfonated PANI (SPANI)-functionalized graphene was stably dispersed in water with an enhancement of the electrochemical stability and electrocatalytic activity [14]. However, simple and effective approaches for fabricating graphene/CP composites remain scientifically challenging.

PANI is a representative conducting polymer for supercapacitor investigation because of its easy synthesis, good environmental stability, doping/dedoping chemistry, high conductivity and excellent capacity for energy storage [7,15,16,17]. However, PANI is susceptible to rapidly reduced performance in specific capacitance during repetitive cycles, due to its swelling and shrinkage [18]. In order to ease this impediment, the incorporation of carbon-based materials in the PANI has been found to enhance the stability and the specific capacitance value at the same time. Recently, graphene/PANI composites with remarkably enhanced performance in supercapacitor electrode materials have been widely investigated due to their conductivity, high surface area and stability for energy storage [6]. Cheng et al. prepared a freestanding and flexible graphene/PANI composite paper via in situ anodic electropolymerization of PANI film on graphene paper with a stable large electrochemical capacitance of 233 F g^−1^ [19]. A hybrid electrode of graphene/PANI composites with an electric double-layer capacitor of graphene nanosheets and a pseudocapacitor of PANI exhibited a synergistic effect with excellent specific capacitance (375.2 F g^−1^) for flexible thin film supercapacitors [20]. However, the specific capacitance in graphene/PANI composites is less provided by the graphene sheets due to its agglomerated layer-like structure and is mainly dominated from the PANI films coated on the graphene sheets [21]. Therefore, it is important to investigate the electrical and electrochemical properties of functionalized graphene to prevent reaggregation [22,23,24,25].

In the previous study, the modified graphene (ADS-G), successfully synthesized through an electrophilic surface modification approach, was dispersed stably in polar solvents and the electrical conductivity was enhanced significantly [26]. In the present work, the ADS-G solution was deposited on nickel foam (the deposited area was about 1 cm × 1 cm) to prepare the functionalized electrode and followed the electrochemical polymerization of aniline to form the functionalized graphene/PANI composites. The electrochemical performances of the ADS-G/PANI composites were studied for further comparison in this article.

## 2. Experimental Section

### 2.1. Materials

Natural graphite flakes were purchased from Sigma-Aldrich (Steinheim, Germany). Aniline was supplied by TCI (C6H7N, ≥98%, Tokyo, Japan). p-Toluenesulfonic acid (PTS) was used as the electrolyte for electrochemical polymerization of aniline (Junsei Chemical Co. Ltd., Tokyo, Japan). Sodium 4-aminoazobenzene-4′-sulfonate (SAS) (TCI, Tokyo, Japan) was applied as a pristine agent to prepare the modifier, ADS. Sodium borohydride and hydrazine monohydrate (TCI, Tokyo, Japan) were used as reducing agents, and potassium permanganate (Sigma-Aldrich, Shanghai, China) was used as oxidizing agent. Sulfuric acid, hydrochloric acid, hydrogen peroxide, and sodium carbonate (Sigma-Aldrich, Shanghai, China) were used as received.

### 2.2. Synthesis of Graphene Xodie and ADS-G

Natural graphite flake was used to prepare graphite oxide by the modified Hummers method [26,27,28]. According to previous research, the ADS-G was synthesized in three steps [26]. The prepared ADS-G was finally dispersed in distilled water and formed the dispersion (1 mg mL^−1^) after sonication for 10-15 min.

### 2.3. Preparation of ADS-G Coated Working Electrode

Prior to the deposition of ADS-G dispersion, the nickel foam was pretreated by etching in 0.1 M HCl solution for 15 min and sonication in acetone and distilled water for 20 min, respectively. The working electrodes were prepared by mixing 1 mL of the obtained ADS-G solution (1 mg mL^−1^), powder with 5 wt.% carbon black and 10 wt.% poly(vinylidene fluoride) (PVDF) binder. A small amount of ethanol was added to the mixture and subsequently sonicated for 30 min to achieve homogeneous dispersion. The resulting dispersion was coated onto the pretreated nickel foam substrate over an area of 1 × 1 cm^2^. The amount of composite on the resulting working electrode including ADS-G, conducting agent (black carbon) and binder (PVDF) was approximately 1.2 mg.

### 2.4. Preparation of ADS-G/PANI Composites

**Figure 1:**
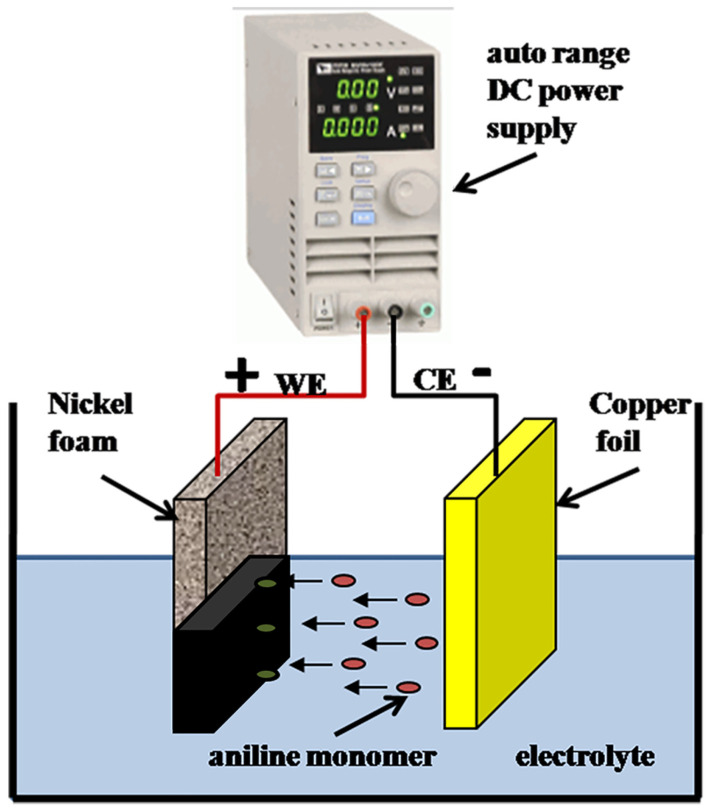
The two electrode configuration for the preparation of ADS-G/PANI composites.

Aniline was freshly distilled under reduced pressure before use. The area of prepared working electrode was used as anodic electrode for the electrochemical polymerization of PANI and the coating area was approximately 1 × 1 cm^2^ for composites [29]. A copper foil (1 × 2 cm^2^) was used as the counter electrode (cathodic electrode). An auto range DC power supply (IT 6721) supplied the power source. The electrochemical polymerization of aniline was executed in the presence of 0.1 M aniline/0.5 M PTS electrolyte solution. The homogeneous mixture of aniline and PTS solution was obtained after sonication for 30 min. The aniline was electrochemically polymerized onto the working electrode at a constant potential (2 V) for 30 min. Subsequently, the working electrode with ADS-G/PANI composites was immersed in 0.5 M H_2_SO_4_ solution to statically remove the aniline monomer and oligomer PANI from the polymeric film and was washed with distilled water and ethanol several times. The resulting electrode was dried at 60 °C in a vacuum for one day. Figure 1 shows the two electrode configurations for the preparation of ADS-G/PANI composites. For comparison, PANI was prepared under the same conditions as the ADS-G/PANI composites.

### 2.5. Characterizations

The chemical characteristics of GO and ADS-G were characterized using Fourier transform infrared spectroscopy (FT-IR) analyses with a Nicolet 6700 spectrometer (Thermo Scientific, Waltham, MA, USA) over the wavenumber range of 4000–400 cm^−1^, and by X-ray photoelectron spectroscopy (XPS) using an AXIS-NOVA analyzer (Kratos Analytical Ltd., Manchester, UK). X-ray diffraction (XRD) patterns, the morphology through a field emission scanning electron microscopy (FE-SEM), and the thermogravimetric analyses (TGA) of the ADS-G, PANI, and ADS-G/PANI composites were recorded precisely; the nickel foam was etched away by a hot HCl or FeCl_3_ solution and then washed with distilled water prior to measurement. The FT-IR analyses of the powder samples of ADS-G, PANI, and ADS-G/PANI composites that were mixed with KBr and pressed into thin pellets were performed with a Nicolet 6700 spectrometer (Thermo Scientific, Waltham, MA, USA) over a wavenumber range of 4000–400 cm^−1^. The XRD patterns of ADS-G, PANI, and ADS-G/PANI composites were obtained on a D/Max 2500V/PC (Rigaku Corporation, Tokyo, Japan) using a Cu target (λ = 0.154 nm) with the scan rate of 2° (2θ) min^−1^, and the range of 5–60°. The morphology of ADS-G, PANI, and ADS-G/PANI composites after etching away the nickel foam and energy-dispersive X-ray spectroscopy (EDS) elemental mapping of ADS-G were observed on an FE-SEM (JSM-6701F, JEOL, Akishima, Japan). The TGA of ADS-G, PANI, and ADS-G/PANI composites were analyzed using a Q50 TG analyzer (TA Instruments, New Castle, DE, USA) from room temperature to 800 °C at a linear heating rate of 5 °C min^−1^ in a nitrogen atmosphere. Electrochemical performances of ADS-G, PANI, and ADS-G/PANI composites electrodes were studied using a CH660D electrochemical workstation. The cyclic voltammograms (CV), and charge/discharge characteristics were operated in a three electrode setup: a platinum foil electrode as the counter electrode, a saturated Ag/AgCl, Cl^−^ electrode as the reference electrode and 6M KOH solution as the electrolyte for all electrochemical measurements.

## 3. Results and Discussion

### 3.1. FT-IR Spectra Analysis

**Figure 2:**
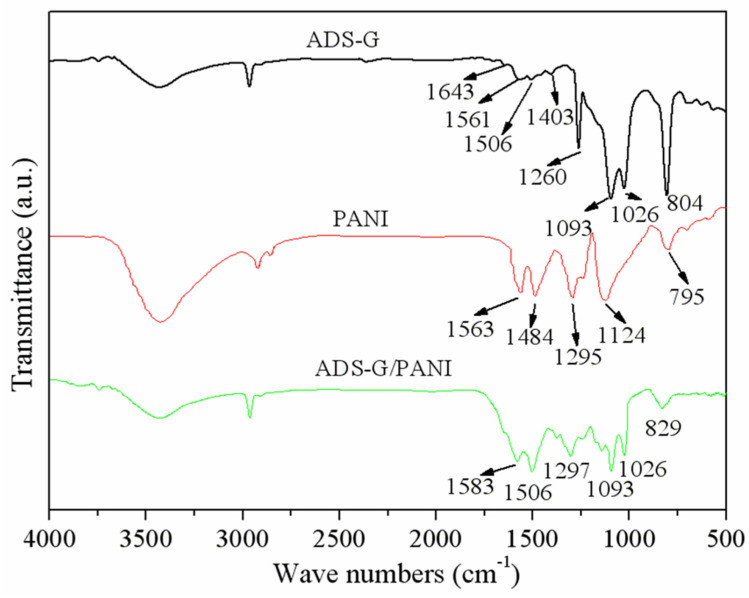
FT-IR spectra of ADS-G, PANI, and ADS-G/PANI composites.

The FT-IR spectra were measured for the ADS-G, PANI, and ADS-G/PANI composites. The peaks at ~698 and 1260 cm^−1^ are shown in Figure 2, suggesting the asymmetric and symmetric stretching vibrations of the S-O and O=S=O groups of -SO_3_^−^. The new peaks of the sulfonic acid group and the C-H out-of-plane vibrations of the benzene ring of ADS molecules were observed at ~1026 and ~804 cm^−1^ [26]. For the PANI powder sample, the absorption peaks appeared at 1563 and 1484 cm^−1^, corresponding to quinoid ring and benzenoid rings in the emeraldine salt, respectively [30]. The appearance of two peaks at 1295 and 1124 cm^−1^ were ascribed to the C-N stretching of the secondary aromatic amine and aromatic C-H bending in the plane [31]. In the case of ADS-G/PANI composites, both of the characteristic peaks of the ADS-G and the PANI were observed in the ADS-G/PANI composites. These findings suggest that the ADS-G/PANI composite was successfully synthesized using electrochemical polymerization.

### 3.2. XPS

**Figure 3:**
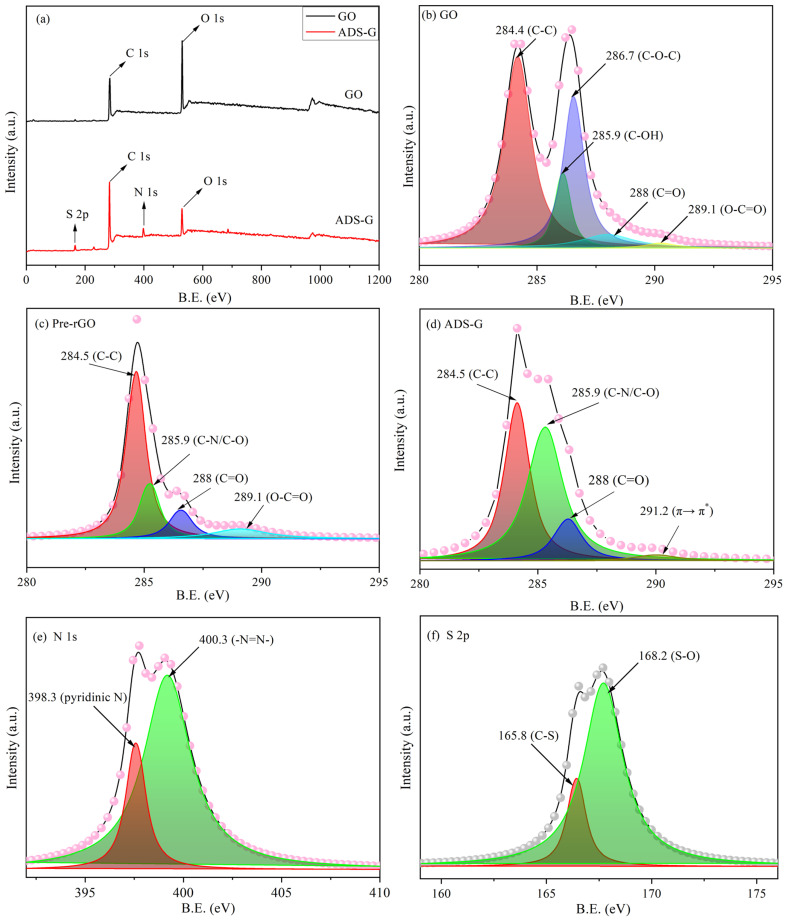
XPS survey of GO and ADS-G (**a**); C1s XPS data of GO (**b**); C1s XPS data of Pre-rGO (**c**) and ADS-G (**d**); N 1s XPS data of ADS-G (**e**); S 2p XPS data of ADS-G (**f**).

XPS analysis was further employed to analyze the elemental characteristics of GO and ADS-G (Figure 3). The XPS surveys of GO and ADS-G are shown in Figure 3a. The peaks at ~167 and 399 eV of S 2p and N 1s were observed for ADS-G, suggesting the ADS molecule was successfully attached to the surface of the graphene sheets. The deconvoluted C 1s XPS of GO (Figure 3b) clearly indicated five components through the various functional groups: the non-oxygenated ring C, the carbon in C-OH, the carbon in C-O-C bonds, the carbonyl C in C=O, and the carboxylate carbon in O-C=O [26,32]. The C 1s XPS of Pre-rGO (Figure 3c) was divided into four components: the non-oxygenated ring C, the C in C-N/C-O, the C in C=O, and the C in O-C=O. In the C 1s XPS of Pre-rGO, the peak intensities of the oxygen functionalities decreased significantly compared to GO and were attributed to the pre-reduction in GO. A unique component of ADS-G was found in the C 1s XPS (Figure 3d): 291.2 eV (π→π* interaction between the ADS and graphene). Compared to GO, the peak intensities of the oxygen functional groups of ADS-G in the C 1s XPS decreased significantly. The two peaks at 398.7 and 400.3 eV of ADS-G were found in the deconvoluted N 1s XPS (Figure 3e), which indicated the nitrogen atoms attached to the aromatic carbon rings belonged to the pyridinic N and the azo (-N=N-) radical groups of ADS-G [33,34]. Two peaks at 165.8 (C-S) and 168.2 (S-O) eV were obviously exhibited in the S 2p XPS (Figure 3f) of ADS-G suggesting the sulfur atoms had bound with ADS-G [35]. All of these observations suggested the existence of ADS on the graphene sheets.

### 3.3. XRD

**Figure 4:**
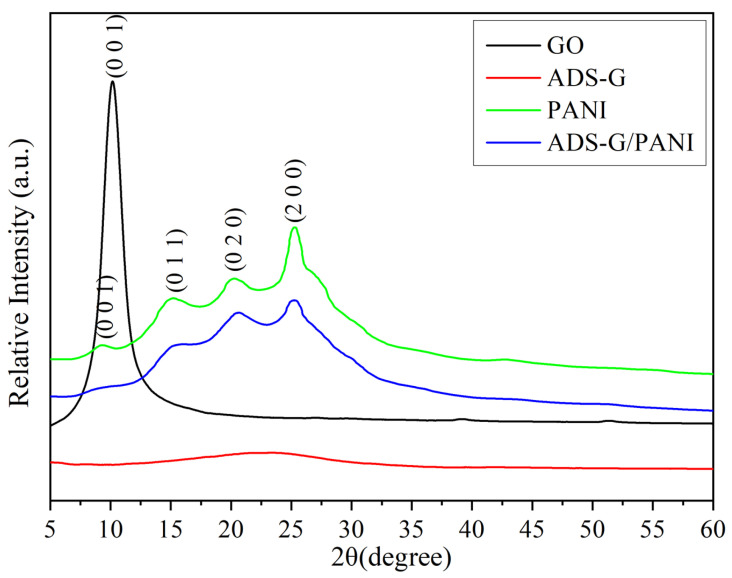
XRD patterns of GO, ADS-G, PANI, and ADS-G/PANI composites.

Figure 4 shows the XRD patterns of GO, ADS-G, PANI, and ADS-G/PANI composites. The representative diffraction peak of GO was observed at approximately 2θ = 10.2°, and the basal reflection (002) peak at 2θ = 26.5°of pristine graphite disappeared in GO [36,37], corresponding to the increased interplanar spacing of the GO sheets, suggesting the intercalation of the new oxygen-containing functional groups and water molecules between the graphite layers [38]. In the case of ADS-G, a very weak peak at 2θ = 23.5° was observed because of the formation of a few agglomerated sheets of graphene and the peak disappeared at 2θ = 10.2°, certifying the reduction in GO, a long-range disorder in graphene, and the complete exfoliation of GO [39]. For PANI specimens, the characteristic peaks appeared at 2θ = 9.4°, 15.1°, 20.5°, and 25.2°, corresponding to (001), (011), (020), and (200) reflections in its emeraldine salt form, respectively [40]. The XRD of the ADS-G/PANI composite exhibited much broader peaks at 2θ = 25.2° and weaker ones at 2θ = 9.4°, 15.1° and 20.5° than those of PANI, indicating that no additional crystalline order was introduced into the ADS-G/PANI composite and ADS-G had interacted with the PANI particles.

### 3.4. FE-SEM Analysis

**Figure 5:**
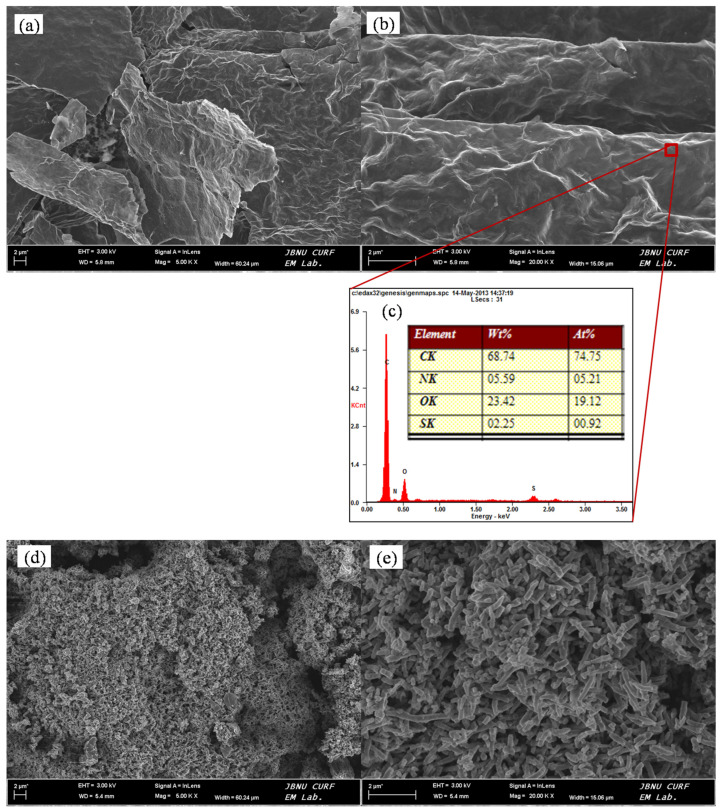

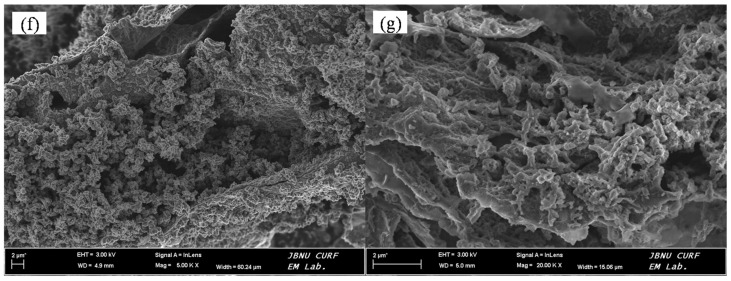
FE-SEM images of ADS-G (**a**,**b**), PANI (**d**,**e**), ADS-G/PANI composites (**f**,**g**) and EDS of ADS-G (**c**); (**a**,**d**,**f**) low-magnification at 5000 and (**b**,**e**,**g**) high-magnification at 20,000.

The morphologies of the prepared ADS-G, PANI, and ADS-G/PANI composites were observed by FE-SEM. Figure 5 shows the FE-SEM images of ADS-G, PANI, ADS-G/PANI composites, and the EDS of ADS-G, respectively. Figure 5a,b show the thin wrinkled flakes suggesting the high surface area of ADS-G after etching away from the nickel foam electrode. From the EDS analysis (Figure 5c), the characteristic peaks for nitrogen and sulfur were clearly found, and typical peaks for nickel were not observed in the ADS-G sheets. This result indicated the ADS-G sheets were fully etched away from the nickel foam. During the electrochemical polymerization of PANI without ADS-G-supported material, the PANI displayed a worm-like morphology with a diameter of about 100–150 nm and a length of 1–2 μm which was stacked by spherical particles in Figure 5d,e. Compared to the morphologies of the ADS-G and PANI, Figure 5f,g show obviously different morphologies of the ADS-G/PANI composites. In the images, the PANI particles grew on the surfaces of the ADS-G, which was like a support material that could provide a large number of active sites for the nucleation of PANI [18,41,42]. This morphology of ADS-G/PANI composites could increase both the dispersion of PANI and the contact of PANI with the electrolyte, and, therefore, may enhance the electrochemical performance as an electrode for a supercapacitor [41].

### 3.5. TGA

**Figure 6:**
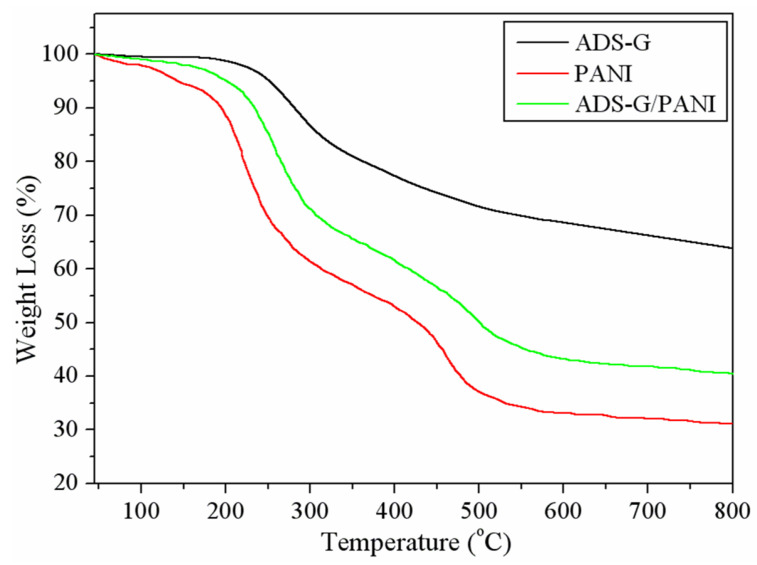
TGA curves of ADS-G, PANI, and ADS-G/PANI composites.

The TGA of ADS-G, PANI, and ADS-G/PANI composites are shown in Figure 6. The significant mass loss of ADS-G took place at approximately 220 °C due to the thermal decomposition process of the PhSO_3_H group and the elimination of residual oxygen functional groups on the surface of the ADS-G [26]. The as-prepared PANI was thermally unstable and showed slight mass loss below 150 °C due to the absorbed moisture in the PANI particles. Subsequently, the PANI exhibited a two-step weight loss. The first main mass loss observed between 150 and 300 °C may be due to the loss of dopant, impurities, and monomer. The second major mass loss was at approximately 400 °C, corresponding to the degradation of the PANI [43]. However, compared to the PANI, the ADS-G/PANI composites showed enhanced thermal stability, indicating the existence of thermally stable ADS-G. The overall mass losses of PANI and ADS-G/PANI composites at 800 °C were approximately 68.91 and 59.59%, respectively, indicating approximately 9.3% ADS-G introduced into the ADS-G/PANI composites. The improved thermal stability of the ADS-G/PANI composites compared to that of PANI may be attributed to the interaction between the ADS-G and PANI chains.

### 3.6. Electrochemical Properties

The electrochemical measurements were taken to demonstrate the structural advantages and further explore the potential applications of the materials in a three-electrode system. The specific capacitance of the electrode can be calculated according to the equation: C = (∫IdV)/(νmV), where I is the response current density (A cm^−2^), V is the potential (V), ν is the potential scan rate (mV s^−1^) and m is the mass of the materials in the electrodes (g).

**Figure 7:**
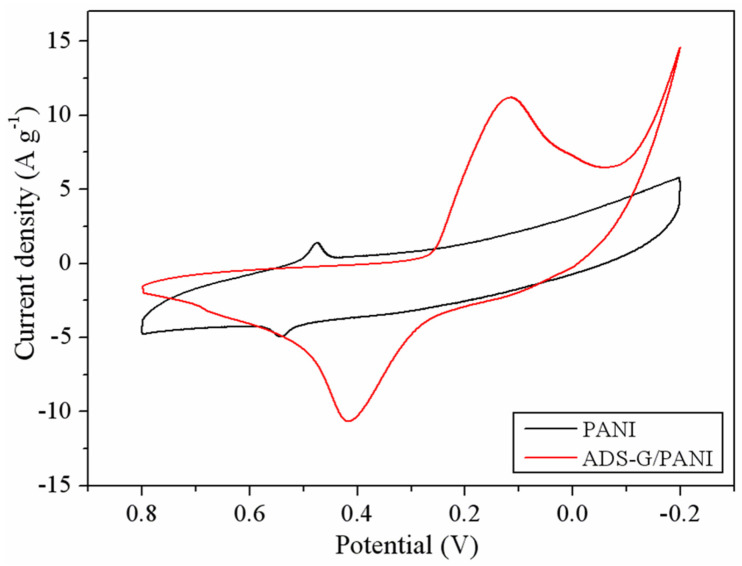
CV curves of PANI and ADS-G PANI composites at a scan rate of 50 mV s^−1^ in 6M KOH.

Figure 7 depicts the CV of PANI and ADS-G/PANI composites at a scan rate of 50 mV s^−1^ in 6M KOH solution. A couple of redox peaks were observed for the PANI and ADS-G/PANI composites in the CV curves, which can be attributed to the redox transitions of PANI crystals, including the redox transformation between a semiconducting state (leucoemeraldine form) and a conducting state (emeraldine form), which indicated the typical pseudocapacitive characteristic of PANI [12]. The area surrounded by the CV curve of ADS-G/PANI composites was apparently larger than that of the PANI at the same scan rate, indicating higher specific capacitance.

**Figure 8:**
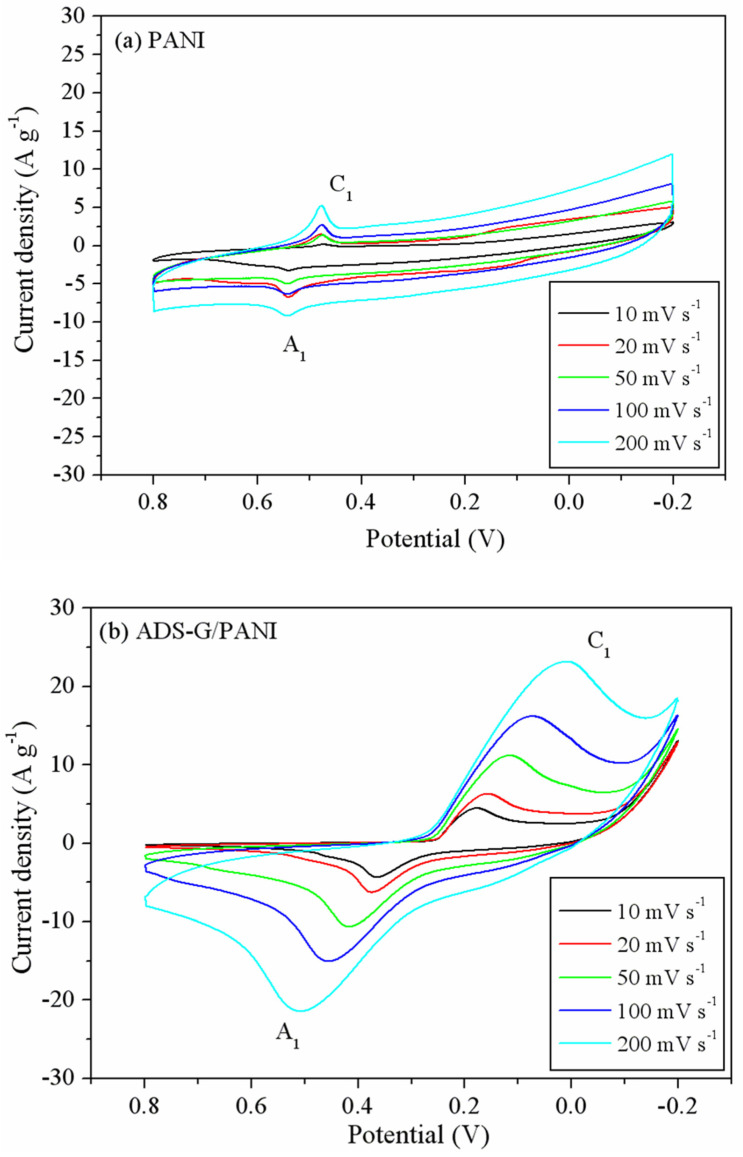
CV curves of (**a**) PANI and (**b**) ADS-G/PANI at various scan rates.

Figure 8 shows the CV curves for the PANI and ADS-G/PANI composites. The CV curves (Figure 8a) of the PANI exhibited a combination of EDLC and pseudocapacitance behavior. However, the shapes of the CV curves for ADS-G/PANI showed more pseudocapacitance behavior than EDLC behavior (Figure 8b). The redox peaks of the PANI and ADS-G/PANI composites are attributed to the faradic transformation of PANI from leucoemeraldine form to emeraldine form. It was also noted that the cathodic peaks (C_1_) shifted negatively and the anodic peaks (A_1_) shifted positively in both the PANI and the ADS-G/PANI composites with increasing potential scan rates from 10 mV s^−1^ to 200 mV s^−1^, which were mainly due to the resistance of the electrode [12]. The area under the CV curves of both the PANI and ADS-G/PANI composites became broader and remained undistorted with the increasing scan rate, indicating excellent supercapacitor behavior up to a high scan rate. And the ADS-G/PANI showed better supercapacitor behavior than that of the PANI, which may be due to the ADS-G-coated nickel-foam electrode serving as the high surface area support material for the polymerization of the PANI and ADS-G in the composites also offering a highly conductive path [44]. The as-prepared ADS-G/PANI composites could provide enhanced electrode/electrolyte interface areas that can enhance the electrochemical accessibility of the electrolyte.

**Figure 9:**
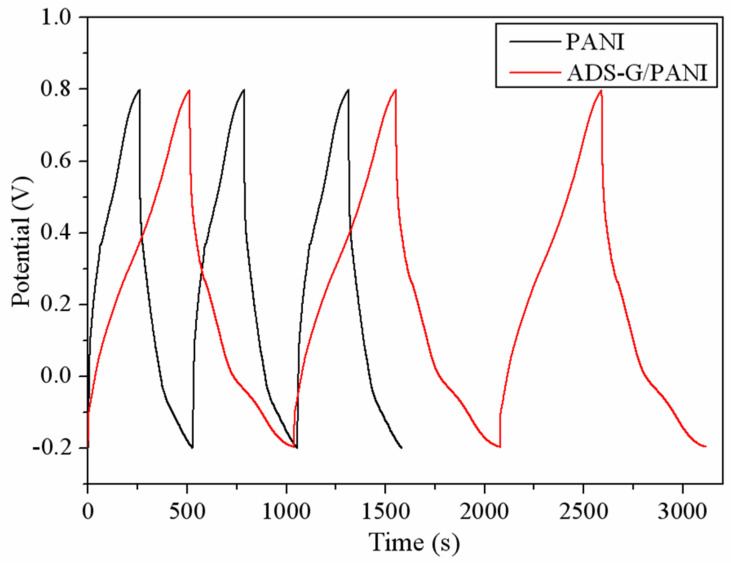
Charge–discharge behavior of PANI and ADS-G/PANI composite-based supercapacitors performed at a constant current density of 1 A g^−1^.

In order to evaluate the specific capacitance of the PANI and ADS-G/PANI composites, the charge/discharge curves were obtained as shown in Figure 9. The charge/discharge cycles of the PANI and ADS-G/PANI composite electrodes were executed at a constant current density of 1 A g^−1^. The specific capacitance can be calculated according to equation C = IΔt/(mΔV), where C (F g^−1^) is the specific capacitance of the materials, I (A) is the applied current, t (s) is the discharge time, V (V) is the applied potential and m (g) is the mass of PANI or ADS-G/PANI composites coated on the nickel electrode. The ADS-G/PANI composites displayed a specific capacitance of 528 F g^−1^ compared to that of 266 F g^−1^ for PANI. The better specific capacitance of the ADS-G/PANI composites compared to that of the PANI is due to the excellent electrode–electrolyte interaction and the synergistic effect of the electrical conductivity between ADS-G and PANI in the composites. By comparing the data in Table 1, it is evident that the ADS-G/PANI composite demonstrates a favorable specific capacitance, surpassing that of similar electrode materials.

**Figure 10:**
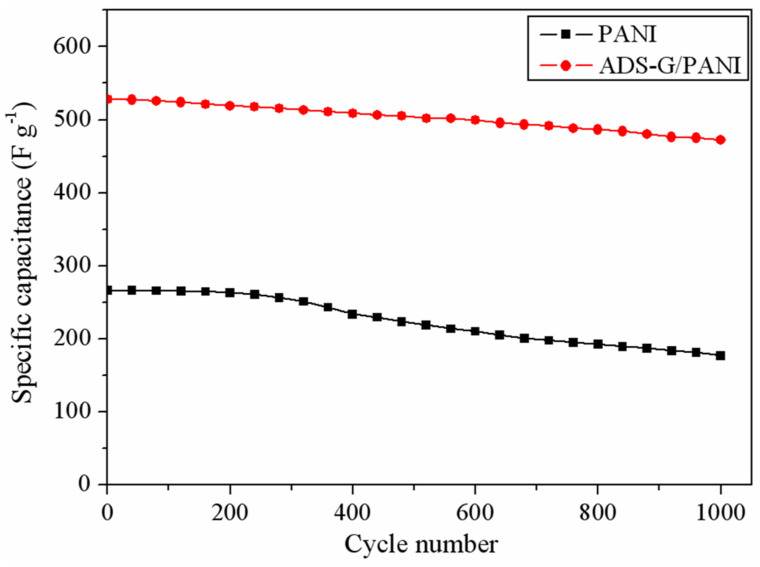
Cyclic stability of PANI and ADS-G/PANI composites recorded at 1 A g^−1^.

Figure 10 shows the cyclic stability of the PANI and the ADS-G/PANI composites. From the image, it can be seen that the ADS-G/PANI composite maintained a specific capacitance of 89.4% of the original value after 1000 charge/discharge cycles, which showed a much better cyclic stability than that of the PANI (66.3%). The high specific capacitance and good cyclic stability of the ADS-G/PANI composite supercapacitor might result from the improved coverage structure and higher conductivity of the ADS-G/PANI composites (Figure 9) compared to that of the PANI.

The power density and the energy density were also used to characterize the electrochemical performance of the as-prepared materials, which can be estimated from the following equations: E = 1/2C(△V)^2^ and P = E/t, where E, C, △V, P and t indicate the average energy density (W h kg^−1^), specific capacitance based on the mass of the ADS-G/PANI composite in the electrodes (F g^−1^), the potential window of discharge (V), average power density (W kg^−1^) and discharge time (s), respectively [12]. The energy densities can reach 73 W h kg^−1^ at a power density of 499 W kg^−1^, which suggests that such ADS-G/PANI composites are quite promising electrode materials for supercapacitors.

## 4. Conclusions

The ADS-G coating on nickel foam was further studied as a high-surface-area support material for the electrochemical polymerization of PANI. The PANI particles were successfully grown on the surface of ADS-G sheets as evidenced by FT-IR and XPS analysis. The morphologies of the ADS-G, PANI, and ADS-G/PANI composites after etching away the nickel foam from the electrodes were proved through FE-SEM analysis. From the FE-SEM and EDS analyses, the large area of the ADS-G for the support materials was revealed. The TGA suggested the incorporation of ADS-G with PANI enhanced the thermal stability compared to that of the PANI alone. The electrochemical performances of both the ADS-G/PANI composites and PANI highlighted their suitability as supercapacitor electrode materials. From the charge/discharge curves, the specific capacitance of the ADS-G/PANI composites was about 528 F g^−1^, which was higher than that of PANI (266 F g^−1^) due to the excellent electrode–electrolyte interaction and synergistic effect of electrical conductivity between the ADS-G and PANI in the composites. The energy densities reaching 73 W h kg^−1^ at a power density of 499 W kg^−1^ were shown by ADS-G/PANI composites. These results suggest that ADS-G/PANI is a suitable composite for potential supercapacitor applications.

## Figures and Tables

**Table 1:** Comparison of electrochemical performance of ADS-G/PANI with other similar electrode materials.

Sample	Current Density (A g^−1^)	Capacitance (F g^−1^)	Electrolyte	Refs
ADS-G/PANI	1	528	6M KOH	This work
PANI-doped graphene	0.1	480	2M H_2_SO_4_	[21]
Graphene-PANI	0.4	475	1M H_2_SO_4_	[30]
Honeycomblike mesoporous carbons (HPCs)	1	339	6M KOH	[45]
PPy/GNS	0.5	482	1M H_2_SO_4_	[46]
Fluoro-functionalized graphene oxide (GOF)/polyaniline (PANI)	1	502	1M H_2_SO_4_	[47]

## Data Availability

Data are contained within the article.

## References

[1] Novoselov K.S., Geim A.K., Morozov S.V., Jiang D., Zhang Y., Dubonos S.V., Grigorieva I.V., Firsov A.A. (2004). Electric field effect in atomically thin carbon films. Science.

[2] Geim A.K., Novoselov K.S. (2007). The rise of graphene. Nat. Mater..

[3] Novoselov K.S., Geim A.K., Morozov S.V., Jiang D., Katsnelson M.I., Grigorieva I.V., Dubonos S.V., Firsov A.A. (2005). Two-dimensional gas of massless dirac fermions in graphene. Nature.

[4] Li D., Kaner R.B. (2008). Graphene-based materials. Science.

[5] Bunch J.S., Zande A.M.V.D., Verbridge S.S., Frank L.W., Tanenbaum D.M., Parpia J.M., Craighead H.G., McEuen P.L. (2007). Electromechanical resonators from graphene sheets. Science.

[6] Choi H.J., Jung S.M., Seo J.M., Chang D.W., Dai L.M., Baek J.B. (2012). Graphene for energy conversion and storage in fuel cells and supercapacitors. Nano Energy.

[7] Ryu K.S., Kim K.M., Park N.G., Park Y.J., Chang S.H. (2002). Symmetric redox supercapacitor with conducting polyaniline electrodes. J. Power Sources.

[8] Faverolle F., Attias A.J., Bloch B. (1998). Highly conducting and strongly adhering polypyrrole coating layers deposited on glass substrates by a chemical process. Chem. Mater..

[9] Lota K., Khomenko V., Frackowiak E. (2004). Capacitance properties of poly(3,4-ethylenedioxythiophene)/carbon nanotubes composites. J. Phys. Chem. Solids.

[10] Snook G.A., Kao P., Best A.S. (2011). Conducting-polymer-based supercapacitor devices and electrodes. J. Power Sources.

[11] Tang Y.H., Wu N., Luo S.L., Liu C.B., Wang K., Chen L.Y. (2012). One-step electrodeposition to layer-by-layer graphene-conducting-polymer hybrid films. Macromol. Rapid Commun..

[12] Yan J., Wei T., Shao B., Fan Z.J., Qian W.Z., Zhang M.L., Wei F. (2010). Preparation of a graphene nanosheet/polyaniline composite with high specific capacitance. Carbon.

[13] Bose S., Kuila T., Uddin M.E., Kim N.H., Lau A.K.T., Lee J.H. (2010). In-situ synthesis and characterization of electrically conductive polypyrrole/graphene nanocomposites. Polymer.

[14] Bai H., Xu Y.X., Zhao L., Li C., Shi G.Q. (2009). Non-covalent functionalization of graphene sheets by sulfonated polyaniline. Chem. Commun..

[15] Li D., Huang J.X., Kaner R.B. (2009). Polyaniline nanofibers: A unique polymer nanostructure for versatile applications. Acc. Chem. Res..

[16] Bai H., Shi G.Q. (2007). Gas sensors based on conducting polymers. Sensors.

[17] Lee R.H., Lai H.H., Wang J.J., Jeng R.J., Lin J.J. (2008). Self-doping effects on the morphology, electrochemical and conductivity properties of self-assembled polyanilines. Thin Solid Films.

[18] Li J., Xie H., Li Y., Liu J., Li Z.X. (2011). Electrochemical properties of graphene nanosheets/polyaniline nanofibers composites as electrode for supercapacitors. J. Power Sources.

[19] Wang D.W., Li F., Zhao J.P., Ren W.C., Chen Z.G., Tan J., Wu Z.S., Gentle l., Lu G.Q., Cheng H.M. (2009). Fabrication of graphene/polyaniline composite paper via in situ anodic electropolymerization for high-performance flexible electrode. ACS Nano.

[20] Lee T.M., Yun T.Y., Park B.H., Sharma B., Song H.K., Kim B.S. (2012). Hybrid multilayer thin film supercapacitor of graphene nanosheets with polyaniline: Importance of establishing intimate electronic contact through nanoscale blending. J. Mater. Chem..

[21] Zhang K., Zhang L.L., Zhao X.S., Wu J.S. (2010). Graphene/polyaniline nanofiber composites as supercapacitor electrodes. Chem. Mater..

[22] Si Y.C., Samulski E.T. (2008). Synthesis of water soluble graphene. Nano Lett..

[23] Kuila T., Khanra P., Bose S., Kim N.H., Ku B.C., Moon B.H., Lee J.H. (2011). Preparation of water—Dispersible graphene by facile surface modification of graphite oxide. Nanotechnology.

[24] Li F.H., Bao Y., Chai J., Zhang Q.X., Han D.X., Niu L. (2010). Synthesis and application of widely soluble graphene sheets. Langmuir.

[25] Hao R., Qian W., Zhang L.H., Hou Y.L. (2008). Aqueous dispersions of TCNQ-anion-stabilized graphene sheets. Chem. Commun..

[26] Yu D.S., Kuila T., Kim N.H., Khanra P., Lee J.H. (2013). Effects of covalent surface modifications on the electrical and electrochemical properties of graphene using sodium 4-aminoazobenzene-4′-sulfonate. Carbon.

[27] Hummers W.S., Offeman R.E. (1958). Preparation of graphitic oxide. J. Am. Chem. Soc..

[28] Kuila T., Bose S., Khanra P., Mishra A.K., Kim N.H., Lee J.H. (2012). A green approach for the reduction of graphene oxide by wild carrot root. Carbon.

[29] Ramkumar R., Sundaram M.M. (2016). Electrochemical synthesis of polyaniline crosslinked NiMoO_4_ nanofibre dendrites for energy storage devices. New J. Chem..

[30] Zhao Y., Tang G.S., Yu Z.Z., Qi J.S. (2012). The effect of graphite oxide on the thermoelectric properties of polyaniline. Carbon.

[31] Yan X.B., Chen J.T., Yang J., Xue Q.J., Miele P. (2010). Fabrication of free-standing, electrochemically active, and biocompatible graphene oxide–polyaniline and graphene–polyaniline hybrid papers. ACS. Appl. Mater. Interfaces.

[32] Stankovich S., Piner R.D., Chen X.Q., Wu N.Q., Nguyen S.B.T., Ruoff R.S. (2006). Stable aqueous dispersions of graphitic nanoplatelets via the reduction of exfoliated graphite oxide in the presence of poly(sodium 4-styrenesulfonate). J. Mater. Chem..

[33] Sharma L., Matsuoka T., Kimura T., Matsuda H. (2002). Investigation into the surface relief grating mechanism via XPS in new azobenzene based optical material. Polym. Adv. Technol..

[34] Feng Y.Y., Liu H.P., Luo W., Liu E.Z., Zhao N.Q., Yoshino K., Feng W. (2013). Covalent functionalization of graphene by azobenzene with molecular hydrogen bonds for long-term solar thermal storage. Sci. Rep..

[35] Kuila T., Khanra P., Kim N.H., Choi S.K., Yun H.J., Lee J.H. (2013). One-step electrochemical synthesis of 6-amino-4-hydroxy-2-napthalene-sulfonic acid functionalized graphene for green energy storage electrode materials. Nanotechnology.

[36] Liu X., Wang X.Y., He P.Y., Yi L.H., Liu Z.L., Yi X. (2012). Influence of borohydride concentration on the synthesized Au/graphene nanocomposites for direct borohydride fuel cell. J. Solid State Electrochem..

[37] Machado B.F., Serp P. (2012). Graphene-based materials for catalysis. Catal. Sci. Technol..

[38] Moon I.K., Lee J.H., Ruoff R.S., Lee H.Y. (2010). Reduced graphene oxide by chemical graphitization. Nat. Commun..

[39] Kaniyoor A., Baby T.T., Ramaprabhu S. (2010). Graphene synthesis via hydrogen induced low temperature exfoliation of graphite oxide. J. Mater. Chem..

[40] Pouget J.P., Józefowicz M.E., Epstein A.J., Tang X., MacDiarmid A.G. (1991). X-ray structure of polyaniline. Macromolecules.

[41] Zhang J.T., Zhao X.S. (2012). Conducting polymers directly coated on reduced graphene oxide sheets as high-performance supercapacitor electrodes. J. Phys. Chem. C.

[42] Zhu J.H., Chen M.J., Qu H.L., Zhang X., Wei H.G., Luo Z.P., Colorado H.A., Wei S.Y., Guo Z.H. (2012). Interfacial polymerized polyaniline/graphite oxide nanocomposites toward electrochemical energy storage. Polymer.

[43] Haldorai Y., Nguyen V.H., Shim J.J. (2011). Synthesis of polyaniline/Q-CdSe composite via ultrasonically assisted dynamic inverse emulsion polymerization. Colloid Polym. Sci..

[44] Wang H.L., Hao Q.L., Yang X.J., Lu L.D., Wang X. (2010). Effect of graphene oxide on the properties of its composite with polyaniline. ACS Appl. Mater. Interfaces.

[45] Guan T.T., Zhao J.H., Zhang G.L., Wang J.L., Zhang D.D., Li K.X. (2019). Template-free synthesis of honeycomblike porous carbon rich in specific 2–5 nm mesopores from a pitch-based polymer for a high-performance supercapacitor. ACS Sustain. Chem. Eng..

[46] Zhang D.C., Zhang X., Chen Y., Yu P., Wang C.H., Ma Y.W. (2011). Enhanced capacitance and rate capability of graphene/polypyrrole composite as electrode material for supercapacitors. J. Power Sources.

[47] Li Y.N., Xing R.G., Zhang B.W., Bulin C. (2019). Fluoro-functionalized graphene oxide/polyaniline composite electrode material for supercapacitors. Polym. Polym. Compos..

